# Clinical efficacy and safety of different tacrolimus concentrations for the treatment of patients with idiopathic membranous nephropathy

**DOI:** 10.1038/s41598-021-92678-y

**Published:** 2021-06-24

**Authors:** Qi Wang, Manna Li, Xuexin Cheng, Gaosi Xu

**Affiliations:** 1 Department of Nephrology, Donghu District, the Second Affiliated Hospital of Nanchang University, Address: No. 1, Minde Road, Nanchang, 330006 People’s Republic of China; 2 Donghu District, the Second Affiliated Hospital of Nanchang University, Address: No. 1, Minde Road, Nanchang, 330006 People’s Republic of China

**Keywords:** Kidney, Kidney diseases

## Abstract

This retrospective study aimed to explore the relative efficacy and safety of different tacrolimus (TAC) concentrations in the treatment of patients with idiopathic membranous nephropathy (IMN). A total of 260 IMN patients with nephrotic syndrome (NS) were recruited. Among these, 125 patients had TAC concentrations no greater than 5 ng/ml (C_TAC_ ≤ 5 ng/ml), and 135 patients had TAC concentrations greater than 5 ng/ml (C_TAC_ > 5 ng/ml). The primary outcomes included complete remission (CR) rates and overall (OR) response rates. The secondary outcomes included 24-h urinary protein (24-h UP), serum albumin and serum creatinine, and adverse events (AEs). During the 12-month follow-up, the overall response rates were significantly different between the C_TAC_ ≤ 5 ng/ml group and the C_TAC_ > 5 ng/ml group (*P* < 0.0001). However, there was no significant difference in the CR at 12 months between the two groups (chi-square, 62% vs 63%, *P* = 0.852). Compared with the C_TAC_ ≤ 5 ng/ml group, the C_TAC_ > 5 ng/ml group had improved levels of 24 h UP (*P* = 0.017) and serum albumin (*P* = 0.010). Moreover, the incidences of acute reversible nephrotoxicity (*P* < 0.001), hepatotoxicity (*P* = 0.036), new-onset diabetes mellitus (*P* = 0.036), and glucose intolerance (*P* = 0.005) were lower in the C_TAC_ ≤ 5 ng/ml group than in the C_TAC_ > 5 ng/ml group. The C_TAC_ > 5 ng/ml group was improved relative to the C_TAC_ ≤ 5 ng/ml group in terms of a PR and CR at 6 months, but there was no difference in the CR between the two groups at 12 months.

## Introduction

Idiopathic membranous nephropathy (IMN) is one of the major causes of adult-onset nephrotic syndrome^1^. In China, because of environmental factors and the aging of the population, the proportion of patients with IMN increased from 6.48% in 1997–1999 to 22.79% in 2009–2011^2^. Among patients with IMN, approximately 80% develop nephrotic syndrome^3^; fortunately, approximately 20% of these patients spontaneously resolve^4^. Unfortunately, approximately 30–40% of IMN patients develop end-stage renal disease (ESRD) within 10–15 years^5^.

There are many treatment programmes for IMN patients, such as glucocorticoids (GC) combined with cyclophosphamide (CTX)^6,7^, GC combined with tacrolimus (TAC), and rituximab^8,9^. However, due to their efficiency and side effects, views on the appropriate treatment of IMN vary. The 2020 KDIGO guidelines recommend TAC for the treatment of IMN. In comparison with no immunosuppressant therapy, TAC monotherapy was recently suggested to be a useful therapeutic option for patients with IMN in a placebo-controlled, randomized trial^7^.

The immunosuppressant TAC (3 ~ 8 ng/ml) is suggested as the first-line treatment for MN in the 2020 KDIGO. However, the 2020 KDIGO does not specify an exact therapeutic concentration, and the definite relationship between serum TAC levels and drug efficacy is not clear. If high and low TAC levels have the same drug efficacy, low TAC levels should be selected to reduce the dose of medication and thereby reduce the side effects. Thus, the primary aim of our study was to explore the efficacy of different TAC levels in IMN patients. In addition, it is uncertain whether adverse reactions to TAC increase with an increasing TAC concentration. TAC doses would need to be selected more cautiously if higher concentrations cause more adverse reactions. We believe that our study is important because it represents the first retrospective and observational study to explore the effectiveness and safety of different doses of TAC for the treatment of IMN patients.

## Methods

### Ethics committee statement

This study was approved by the Regional Ethics Committee of the Second Affiliated Hospital of Nanchang University, and the approval number was No. [2020] 026. Informed consent was waived by the Ethics Committee of the Second Affiliated Hospital of Nanchang University. The design of the study took the safety and fairness principles fully into account. This research did not harm the subjects and protected their privacy. There were no conflicts of interest in this research.

### Patients

A retrospective cohort study was carried out in 'real-world' conditions via an observational design. Adult (18–70 years) patients with nephrotic syndrome who were treated between March 2014 and April 2019 in the Department of Nephrology, the Second Affiliated Hospital of Nanchang University, Jiangxi Province, China, were eligible for enrolment. Then, we analysed their medical records to identify standard treatment-compliant patients who met the inclusion criteria: IMN proven by renal biopsy; nephrotic syndrome, defined as the presence of nephrotic-range proteinuria > 3.5 g/24 h, serum albumin < 30 g/L, oedema, and/or hyperlipidaemia; eGFR > 60 ml/min per 1.73 m^2^; a serum TAC concentration that was stabilized within a month; no immunosuppressive agents used in the previous 6 months; and a follow-up time of at least 12 months. The exclusion criteria were as follows: serious complications such as thrombosis, renal failure and infection; serious diseases such as HIV, cardiac dysfunction, hepatitis B, hepatitis C or serum amino-transferase exceeding twofold the normal upper limit; other contraindications to immunosuppressants; hypersensitivity to macrolide medication; diabetes mellitus or the coexistence of other severe kidney diseases; pregnancy or lactation; and secondary diseases that cause membranous nephropathy such as systemic lupus erythematosus; patients who during follow-up, or in the periods between two determinations, have presented levels that indicate a change of group.

### Treatment protocol

Among all collected NS patients, one cohort (n = 125) included standard-compliant patients whose blood concentrations of TAC were no greater than 5 ng/ml (C_TAC_ ≤ 5 ng/ml), and the second cohort (n = 135) included standard-compliant patients whose blood concentrations of TAC were greater than 5 ng/ml (C_TAC_ > 5 ng/ml). Therapy with TAC was initiated at a dose of 0.05 ~ 0.075 mg/kg/d (0.5–1 mg capsules) in 2 divided doses before meals at 12 h intervals. The whole blood TAC level was regularly checked (every 1 week during the first 4 weeks and then every 2 weeks for 8 weeks and then monthly during the remaining 9 months) for all TAC users. The trough concentration of TAC in fasting whole blood was measured by a chemiluminescence microparticle immunoassay (CMIA). The TAC concentration was determined after stabilization for at least 4 weeks. Six months later, the dosage of TAC was adjusted by the physician mainly based on the patients' manifestations. For patients who had achieved a complete remission (CR), the TAC was tapered and maintained at the minimum effective dose during the follow-up. For the patients who showed no response at all, TAC was discontinued after 6 months, and they were categorized as no remission (NR) at 12 months. For the rest of the patients, full-dose TAC was continued until a CR or PR was achieved. In addition, a daily dose of oral GC was initiated at 15–20 mg in parallel with tacrolimus every morning.

### Follow-up and outcomes

Follow-up of the participants was scheduled at 6 and 12 months after the initiation of the immunosuppression described above. We predefined the potential baseline indexes, including sex, age, systolic blood pressure, diastolic blood pressure, TAC concentration, 24-h urinary protein (24-h UP) level, serum creatinine (Scr) level, serum albumin level, eGFR, total cholesterol level, triglyceride level, high-density lipoprotein level, low-density lipoprotein level, lipoprotein (mmol/L) level, UA level, fasting glucose level, HbA1c level, and anti-PLA2R. In addition, the use of GCs, statins and angiotensin-converting enzyme inhibitors (ACEIs)/angiotensin receptor blockers (ARBs) were observed in this patient population. Moreover, the use of cyclophosphamide therapies was not allowed.

The primary efficacy assessment was a CR, defined as a 24 h UP level < 0.3 g and the serum albumin had to be ≥ 30 g/l with stable renal function. We also evaluated the rates of an overall response [CR plus partial remission (PR)] at months 6 and 12, with PR defined as reduced no less than 50% of the baseline UP levels plus a final UP < 3.5 g/24 h but > 0.3 g/24 h with a normal or improved serum albumin peak and stable Scr.

The secondary efficacy assessments included 24 h UP, serum albumin, Scr and the incidence of any adverse events (AEs). No remission (NR) was defined as patients who did not achieve the CR or PR criteria after 6 months of TAC. Renal relapse (RR) was defined as an increase in urinary protein excretion > 3.5 g/d in consecutive analyses in patients with CR or PR. Acute reversible nephrotoxicity was defined as an increase in the serum creatinine level greater than 25% compared with baseline, which improved after a 50% reduction in TAC daily dosage for 15 days. Persistent nephrotoxicity was defined as an increase in serum creatinine level greater than 50% compared with baseline, which persisted despite a 50% reduction in the TAC dose after 15 days^10^.

### Statistical analysis

Normally distributed variables are expressed as the mean ± standard deviation, and an independent or paired t-test was used for comparison when appropriate. Nonparametric continuous variables were represented by the median of the quartile interval [(IQR) 25th and 75th percentile] and were compared as appropriate using nonparametric tests. Categorical variables were summarized as proportions and Pearson’s chi-square test was used for comparisons. The time-event data were described by Kaplan–Meier curves, and the differences between the groups were compared by log-rank tests. The longitudinal data analysis method was used to analyse the repeated measures data. GraphPad Prism (Version 7.0, San Diego, USA) and SPSS (Version 23.0, Chicago, USA) were used for statistical analysis. Receiver–operator characteristics (ROC) analysis was used to obtain a representative cut-off value for TAC levels between responders versus non-responders. To obtain this distinct value, a trade-off was made between sensitivity and specificity. Patients were divided into a group of non-responders and a group of responders according to an overall response criterion at months 12. And the differences in the area under the curve (AUC) were analyzed using MedCalc version 15.0 (MedCalc Software, Mariakerke, Belgium). When the bilateral P value < 0.05, the differences were considered to be statistically significant.

## Results

### Patients and baseline characteristics

**Figure 1:**
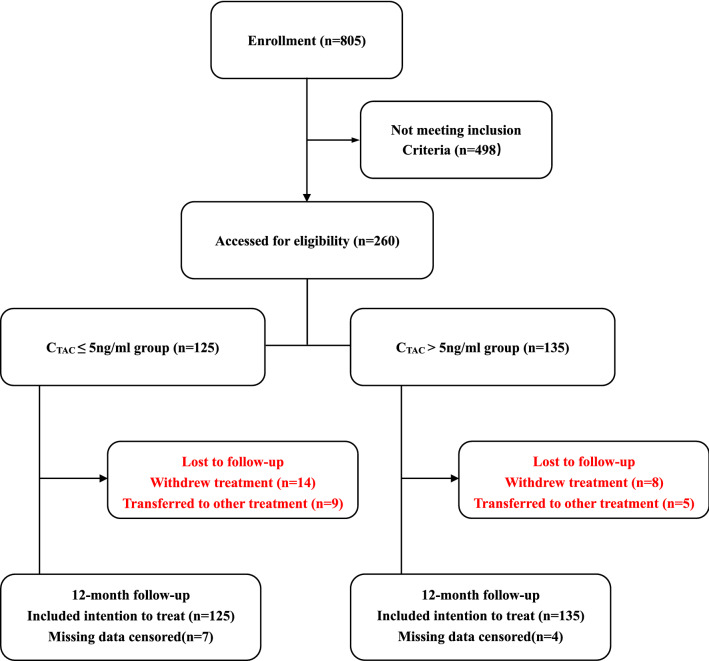
Flow diagram for the inclusion of the participants.

A flowchart for selecting patients is shown in Fig. 1. Preliminary screening identified 805 patients, and 260 subjects were finally included in this study. A total of 125 patients received TAC at serum concentrations of no more than 5 ng/ml, and 135 patients with a similar risk profile with regard to progression received TAC at serum concentrations greater than 5 ng/ml. There were seven censored patients among the standard–compliant patients whose serum TAC concentrations were no more than 5 ng/ml who were missing important data (24 h UP) during follow-up. Among the standard-compliant patients whose concentrations were greater than 5 ng/ml, four lacked important data (24 h UP level and medication administration record) during follow-up. These patients were excluded from the study. At baseline, the patient characteristics were similar between the two cohorts (Table 1).

**Table 1:** Clinical features of participants at baseline.

Characteristics	C_TAC_ ≤ 5 ng/ml (*n* = 125)	C_TAC_ > 5 ng/ml (*n* = 135)	*P* value
Male (%)	79 (63.3)	93 (68.8)	0.315
Asian	125	135	1
Age (y)	46.58 ± 19.53	46.21 ± 17.14	0.149
Systolic blood pressure (mmHg)	126.80 ± 19.76	127.19 ± 18.45	0.624
Diastolic blood pressure (mmHg)	80.85 ± 13.05	81.26 ± 11.64	0.574
Tacrolimus concentration	3.24 ± 1.35	6.74 ± 0.91	0.000
24 h UP (g/24 h)	5.95 ± 5.70	7.39 ± 6.64	0.356
Serum creatinine (μmol/L)	120.38 ± 241.68	93.21 ± 73.09	0.179
Serum albumin (g/L)	28.85 ± 9.56	28.64 ± 8.80	0.532
eGFR (ml/min per 1.73 m^2^)	86.58 ± 35.22	97.17 ± 40.68	0.321
Total cholesterol (mmol/L)	7.24 (4.93 to 8.47)	8.44 (5.49 to 10.42)	0.074
Triglycerides (mmol/L)	3.02 (1.48 to 3.56)	3.07 (1.54 to 3.64)	0.954
High-density lipoprotein (mmol/L)	1.53 (1.16 to 1.79)	1.54 (1.12 to 1.88)	0.348
Low-density lipoprotein (mmol/L)	5.12 (3.24 to 6.62)	5.01 (3.24 to 6.42)	0.825
Lipoprotein (mmol/L)	48.93 ± 40.61	56.56 ± 42.16	0.458
UA (μmol/L)	387.82 ± 107.05	370.69 ± 101.26	0.799
Fasting glucose (mmol/L)	5.61 (4.71 to 5.96)	5.73 (4.38 to 6.40)	0.016
HbA1c (%)	5.56 (5.20 to 5.83)	5.71(5.20 to 5.90)	0.516
Anti-LA2R (positive/negative)	124/1	134/1	0.79
Use of glucocorticoid	106 (85)	119 (88)	0.115
Statin use (%)	35.37	31.25	0.346
Use of ACEI/ARB(%)	65 (52)	74 (55)	0.439

### Effectiveness

**Figure 2:**
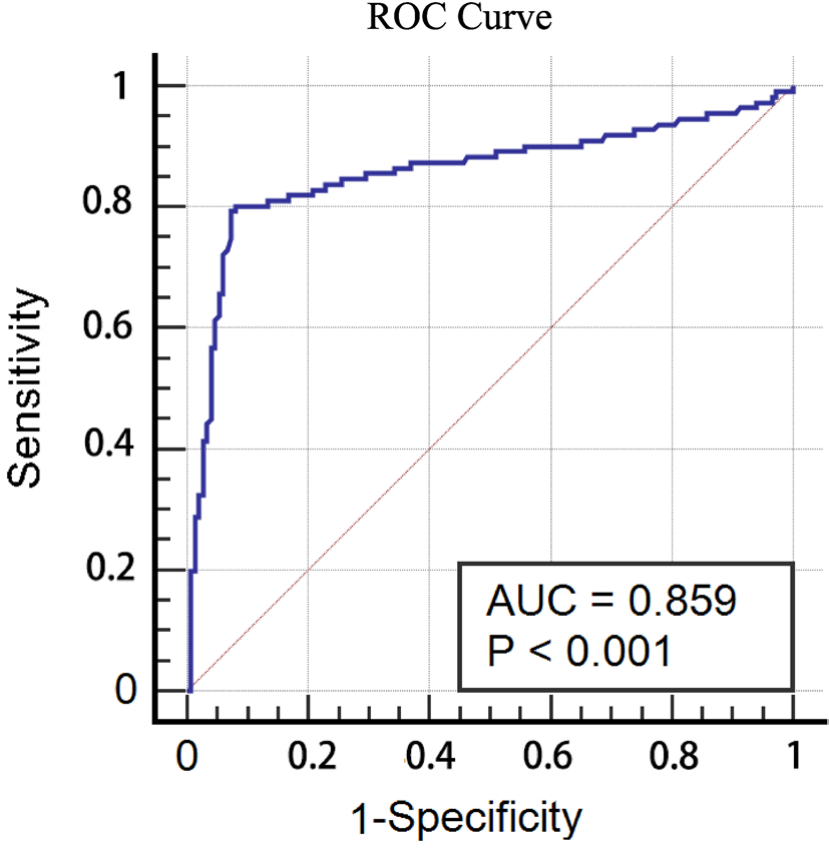
ROC-curve analysis: overall non and response. ROC-curve analysis with trough level concentrations of TAC. To optimally distinguish between overall non versus responders a cut-off value of 5 ng/mL was found with an AUC of 0.859 (95% CI 0.810 to 0.898, *P* < 0.0001), with a specificity of 0% and sensitivity of 80.18%.

**Figure 3:**
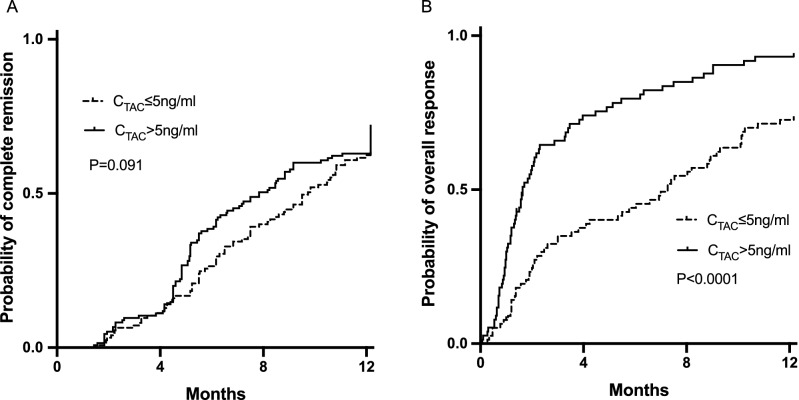
Kaplan–Meier analysis for complete remission rates (**A**) and overall response rates (**B**) in patients with idiopathic membranous nephropathy.

Figure 2 shows an area under the curve (AUC) of 0.859 (95%CI 0.810 to 0.898, *P* < 0.0001). At 5 ng/mL, a sensitivity of 80.18% and a specificity of 0% were found. The AUC in the ROC curve is significantly different from 0.5, concluding that TAC concentration has the ability to distinguish between the group of responders and the group of non-responders. As shown in Table 2, the rates of CR were higher in the C_TAC_ > 5 ng/ml group than in the C_TAC_ ≤ 5 ng/ml group (chi-square, 38% vs 26%, *P* < 0.001) at 6 months. However, there was no significant difference in the CR at 12 months between the two groups (chi-square, 62% vs 63%, *P* = 0.852). The Kaplan–Meier analysis for the probability of a CR was not significantly different between the C_TAC_ ≤ 5 ng/ml group and the C_TAC_ > 5 ng/ml group (*P* = 0.091, Fig. 3A). Figure 3B shows the Kaplan–Meier analysis of the overall responses, and there was a statistically significant difference between the two groups at 12 months (*P* < 0.0001).

**Table 2:** End points on the basis of the available patients at the end of the study phase.

End Point	C_TAC_ < 5 ng/ml (*n* = 125)	C_TAC_ > 5 ng/ml (*n* = 135)	*P* value
	End Point Value	End Point Value	
Primary outcomes			
CR at month 6	33 (26)	51 (38)	0.000
CR at month 12	78 (62)	85 (63)	0.852
Secondary outcomes			
Relapse rates	3 (4)	5 (6)	0.255
No response	17 (14)	18 (13)	0.900
Change in 24 h proteinuria at month 6	− 2.66 (− 5.42 to − 0.00)	− 5.62 (− 10.22 to − 0.04)	0.001
Change in 24 h proteinuria at month 12	− 3.55 (− 7.07 to − 0.00)	− 6.00 (− 10.55 to − 0.50)	0.017
Change in serum albumin at month 6	7.24 (0.54 to 14.25)	8.07 (2.03 to 13.45)	0.023
Change in serum albumin at month 12	9.04 (3.38 to 16.90)	9.98 (3.69 to 16.07)	0.010
Change in Serum creatinine at month 6	4.75 (− 16.00 to18.3)	6.69 (− 12.45 to 11.37)	0.621
Change in Serum creatinine at month 12	12.45 (− 23.1to 24.46)	7.39 (− 17.06 to 24.18)	0.315

### Urinary protein

**Figure 4:**
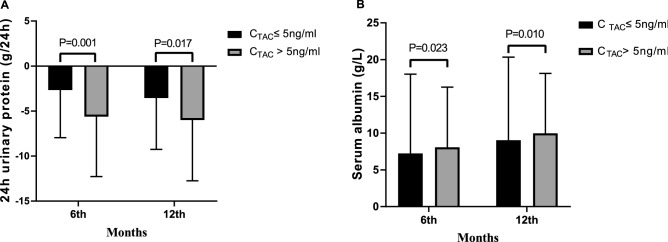
Comparison of the decreased levels of 24 h urinary protein and serum albumin before and after treatment in the two groups. (**A**) Decreased levels of 24 h urinary protein prior to and after treatment in the 2 groups. (**B**) The decreased levels of serum albumin prior to and after treatment in the 2 groups.

In the C_TAC_ ≤ 5 ng/ml group, the 24 h UP level decreased by a median of -2.76 g/L (IQR, -5.42 to -0.00 g/L) at 6 months, and the C_TAC_ > 5 ng/ml group had a remarkably reduced UP by 5.62 (IQR, -10.22 to -0.40 g/L, *P* = 0.001, Table 2, Fig. 4A). At the 12th month of follow-up, proteinuria decreased by a median of 3.65 g/d (IQR, -7.15 to -0.00 g/d) and 6.00 g/d (IQR, -10.55 to -0.50 g/d) in the C_TAC_ ≤ 5 ng/ml group and the C_TAC_ > 5 ng/ml group, respectively (*P* = 0.014, Table 2, Fig. 4A).

### Serum albumin

At the 6-month follow-up, there was a statistically significant difference in serum albumin between the groups (IQR was 0.52 to 14.17 g/l and 2.03 to 13.45 g/l in the C_TAC_ ≤ 5 ng/ml and C_TAC_ > 5 ng/ml groups, respectively) (*P* = 0.021, Table 2, Fig. 4B). At the end of 12 months, the C_TAC_ > 5 ng/ml group had remarkably increased serum albumin compared with the C_TAC_ ≤ 5 ng/ml group. Serum albumin increased by a median of 8.76 g/l (IQR, 3.37 to 16.72 g/l) and 9.98 g/l (IQR, 3.69 to 16.07 g/l) in the C_TAC_ ≤ 5 ng/ml and C_TAC_ > 5 ng/ml groups, respectively (*P* = 0.008, Table 2, Fig. 4B).

### Serum creatinine

The Scr level in the C_TAC_ ≤ 5 ng/ml group decreased by a median of 4.75 μmol/L (IQR, -16.00 to 18.3 μmol/L) at 6 months, and the C_TAC_ > 5 ng/ml group increased by 6.69 μmol/L (IQR, -12.45 to 11.73 μmol/L) (*P* = 0.62, Table 2). There was no significant difference between the two groups regarding the concentration of Scr at the end of the trial (the IQR was -23.1 to 24.46 μmol/L and -17.06 to 24.18 μmol/L in the C_TAC_ ≤ 5 ng/ml and C_TAC_ > 5 ng/ml groups, respectively) (*P* = 0.315, Table 2). There was no end-stage renal failure (ESRF) in any group.

### Adverse events

All adverse events occurring between study inclusion and the last follow-up visit were observed (Table 3). During the 12-month treatment, the main adverse reactions of the two groups of patients included urinary tract infections, bacterial pneumonia, acute reversible nephrotoxicity, persistent nephrotoxicity, hepatotoxicity, gastrointestinal symptoms, new-onset diabetes mellitus, and glucose intolerance. No patient died or progressed to ESRD during the follow-up. Four patients (3%) with diabetes were observed in the C_TAC_ ≤ 5 ng/ml group, and 8 patients (6%) were observed in the C_TAC_ > 5 ng/ml group (*P* = 0.036, Table 3). All of them received oral antidiabetic drugs or insulin treatment, and the blood glucose levels in two patients returned to normal after TAC withdrawal in the C_TAC_ ≤ 5 ng/ml group. In addition, the hazards of glucose intolerance considered separately were approximately two-fold lower in the C_TAC_ ≤ 5 ng/ml group than in the C_TAC_ > 5 ng/ml group (*P* = 0.005, Table 3). The incidence of hepatotoxicity, presented as an elevation of ALT (78–235 IU/l; normal: 7–40 U/l) and AST (86–286 IU/l; normal: 13–35 IU/l), [2% (2/125) versus 4% (5/135) *P* = 0.0036] was significantly lower in the C_TAC_ ≤ 5 ng/ml group than in the C_TAC_ > 5 ng/ml group. Acute reversible or persistent nephrotoxicity was observed in 40 patients. All episodes were observed within the first 2 months of therapy. The incidence of acute reversible nephrotoxicity [8 vs 16% (18/31), *P* < 0.001)] was significantly lower in the C_TAC_ ≤ 5 ng/ml group than in the C_TAC_ > 5 ng/ml group, while there was no significant difference between the two groups regarding persistent nephrotoxicity (*P* = 0.225, Table 3). The differences in the rates of infections, urinary tract infections, and bacterial pneumonia in the two groups were not statistically significant.

**Table 3:** Summary of adverse events (AEs).

Side effects	C_TAC_ ≤ 5 ng/ml (*n* = 125)	C_TAC_ > 5 ng/ml (*n* = 135)	*P* value
Infections	7(7)	8(6)	0.823
Urinary tract infection	3(2)	4(3)	0.577
Bacterial pneumonia	4(3)	4(3)	0.826
^a^Acute reversible nephrotoxicity	18(8)	31(16)	0.000
^b^Persistent nephrotoxicity	3(2)	5(4)	0.225
Hepatotoxicity	2(2)	5(4)	0.036
Gastrointestinal symptoms	0	0	
New-onset diabetes mellitus	4(3)	8(6)	0.036
Glucose intolerance	5(4)	11(8)	0.005

^a^Acute reversible nephrotoxicity: Scr 25% above baseline value, recovered when the dosage of drug decreased.

^b^Persistent nephrotoxicity: Scr 50% above baseline, which persisted despite 50% reduction of TAC dose for 15 days.

## Discussion

Treatment of IMN remains a challenge for nephrologists. Recently, IMN has come to be regarded as a kind of glomerular damage mediated by autoantibodies to the antigenic components of the podocyte membrane^11^. TAC stabilizes the podocyte cytoskeleton by inhibiting the expression of calcine phosphatase and the transient receptor potential cation channel 6 (TRPC6) protein, resulting in reduced urinary protein levels^12,13^. TAC alleviates nephrotic syndrome in the majority (80%) of IMN patients^7,14^. TAC has been reported to be effective in treating IMN patients with either monotherapy or in combination with corticosteroids^15–20^. More importantly, some studies have also shown that TAC plays an important role in corticosteroid-resistant primary glomerulopathy^21,22^. However, none of these studies were concerned about the exact relationship between TAC levels and drug efficacy. Therefore, we believe that our study is important because it represents the first retrospective and observational study to address this knowledge gap. With the identification of this concentration–effect relationship of TAC in patients with MN, new opportunities have emerged to optimise treatment and reduce costs. Although TAC concentrations vary widely between patients, with an AUC of 0.859, a drug level of 5 ng/mL has a predictive value of good clinical response according to the overall response criteria. At 5 ng/mL, a sensitivity of 80.18% and a specificity of 0% were found. Since there are also other reasons for non-response in addition to low drug levels, in other words, there are non-responders with adequate or high drug levels, specificity is not likely to be very high in our population. We compared the efficacy and safety of different serum TAC concentrations in patients with IMN. Although we observed that the C_TAC_ > 5 ng/ml group had remarkably higher overall response rates than the C_TAC_ ≤ 5 ng/ml group at month 6, there was no difference in the treatment outcomes between the two groups at month 12. These results are similar to those of the 2020 KDIGO.

It is very common for patients receiving CNIs to experience nephrotoxicity. After cessation of CNIs, the nephrotoxicity is reversible in most patients. However, a small number of people have irreversible nephrotoxicity and develop chronic kidney disease. After 12 months of treatment, the histologically confirmed incidence of CNI nephrotoxicity (clinical + subclinical) was 76.4% in patients who underwent a biopsy^23^. TAC nephrotoxicity occurred in approximately 25% of the patients in the current study, with an incidence similar to that reported in various other studies^18,24^. Moreover, compared with the C_TAC_ ≤ 5 ng/ml group, acute reversible nephrotoxicity was more likely to occur in the C_TAC_ > 5 ng/ml group. In addition, 0.74% (1/135) of the patients who developed persistent nephrotoxicity were classified as CKD (eGFR < 60 ml/min/1.73m^2^ for 3 months or more) in the C_TAC_ > 5 ng/ml group at the end of the follow-up, while none of the patients developed CKD in the C_TAC_ ≤ 5 ng/ml group at the end of the follow-up.

Furthermore, there was a higher rate of new-onset diabetes mellitus and glucose intolerance in the C_TAC_ > 5 ng/ml group than in the C_TAC_ ≤ 5 ng/ml population. These results are supported by emerging evidence that TAC affects calcineurin, a central signalling controller in eukaryotes^25^, which results in multisystemic side effects such as pathological glycaemia^26,27^. In the secondary outcome measures, the present study found that the C_TAC_ > 5 ng/ml group was superior to the C_TAC_ ≤ 5 ng/ml group for improvements in 24 h proteinuria and serum albumin.

This study had several limitations. First, the course of therapy and follow-up were quite short; thus, the long-term AEs of high concentrations of TAC remain to be investigated. Second, missing data were inevitable. However, they would tend to bias the results towards the null hypothesis. Therefore, more multicentre controlled randomized clinical trials should be conducted in the future to assess the efficacy and safety of different doses of TAC for IMN. In addition, the serum concentration of the TAC may influence the decrease or negativization of anti-PLA2R and at the same time, that this influences the clinical outcomes.

In conclusion, C_TAC_ > 5 ng/ml was more effective than C_TAC_ ≤ 5 ng/ml in terms of the PR and CR in treating IMN patients with NS at 6 months, but there was no difference in the CR between the two groups at 12 months. Since high concentrations of TAC are associated with more AEs, we recommend reducing the concentration of TAC as far as possible to reduce the incidence of AEs while not affecting the patient’s chance of achieving a long-term CR. This conclusion is worthy of further clinical investigation.
